# Supplementary material for: How deep ocean-land coupling controls the generation of secondary microseism Love waves
Source: Nat Commun. 2021 Apr 20;12:2332. doi: 10.1038/s41467-021-22591-5 (PMC8058104; doi:10.1038/s41467-021-22591-5)
Supplement: Supplementary file 1 — Supplementary Information [file 41467_2021_22591_MOESM1_ESM.pdf]

Supplementary information for

How deep ocean-land coupling controls the generation of secondary microseism Love waves

Florian Le Pape[1,2,*], David Craig[1,2] and Christopher J. Bean[1,2]

[1]Geophysics Section, Dublin Institute for Advanced Studies, 5 Merrion Square, Dublin 2, Ireland

[2]iCRAG centre, University College Dublin, Belfield, Dublin 4, Ireland

[*]Corresponding author, email: flepape@cp.dias.ie

SUPPLEMENTARY FIGURES

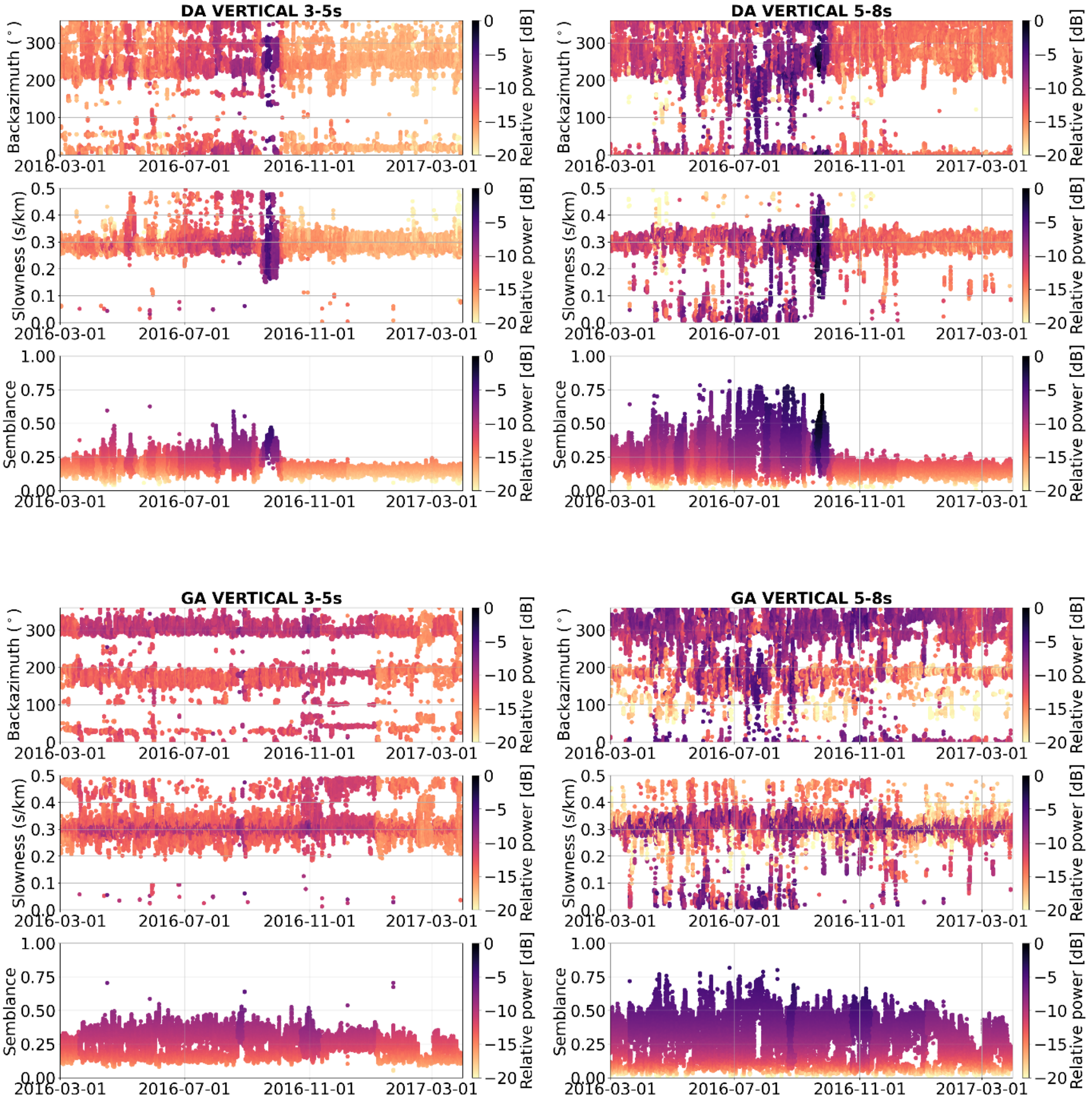

Supplementary Figure 1 - Array analysis of real data (Z component). Back azimuth, slowness and semblance associated with vertical (Z) components of both Donegal (DA) and Galley Head (GA) arrays. For each array, the analysis is performed over a year of data in period bands 3-5s and 5-8s.

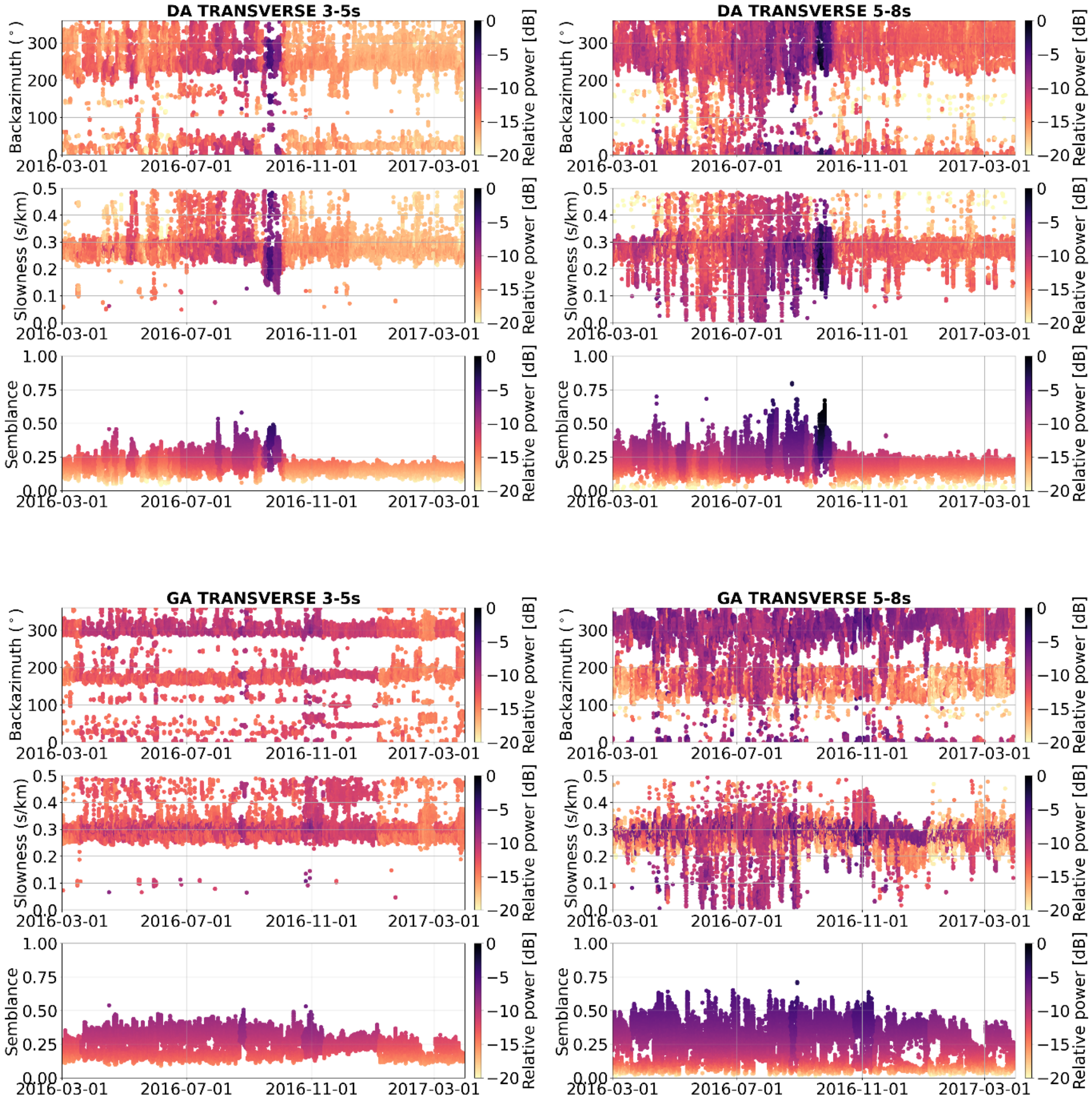

Supplementary Figure 2 - Array analysis of real data (T component). Back azimuth, slowness and semblance associated with transverse (T) components of both Donegal (DA) and Galley Head (GA) arrays. For each array, the analysis is performed over a year of data in period bands 3-5s and 5-8s.

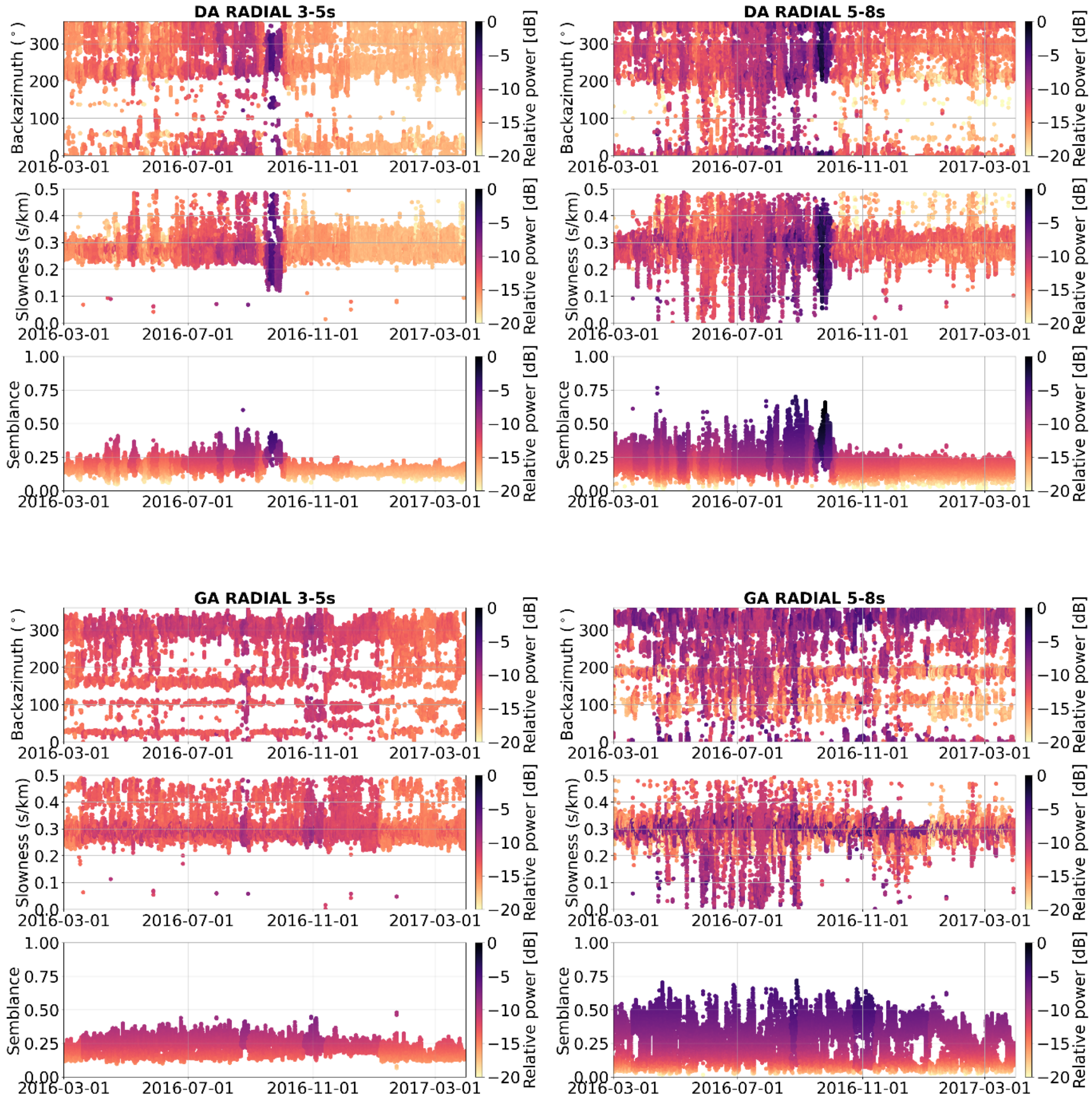

Supplementary Figure 3 - Array analysis of real data (R component). Back azimuth, slowness and semblance associated with radial (R) components of both Donegal (DA) and Galley Head (GA) arrays. For each array, the analysis is performed over a year of data in period bands 3-5s and 5-8s.

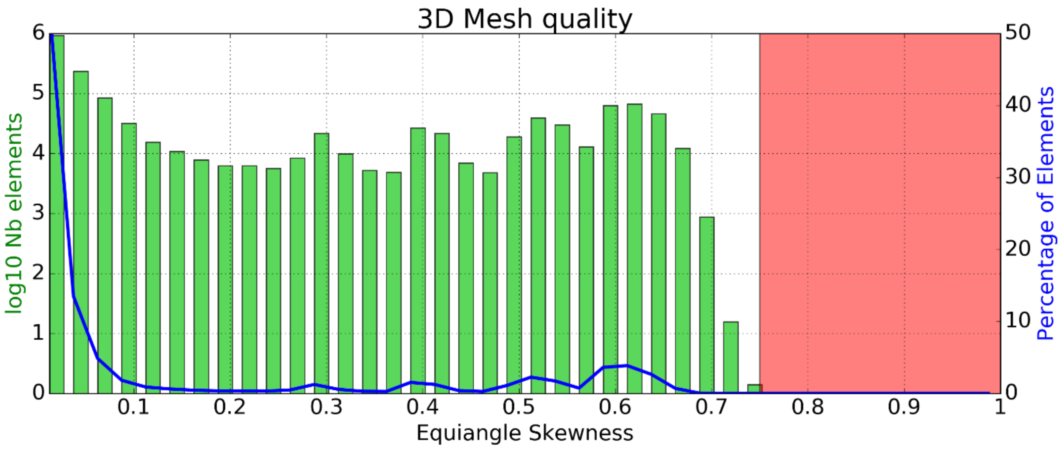

Supplementary Figure 4 - Mesh quality of the Irish offshore 3D model. The model comprises 4,939,533 elements. The blue curve represents the percentage of elements and the green bars the numbers of elements associated with different skewness values between 0 (very good mesh quality) and 1 (very bad mesh quality). The max threshold defined in the SPECFEM3D manual as an acceptable limit for mesh quality is 0.75. Above that value (red area), the elements need to be re-meshed. With only 49 elements above 0.7 skewness (max being 0.728) and more than 75% of elements below 0.3, the mesh quality is considered acceptable due to the complexity of the model over a wide area.

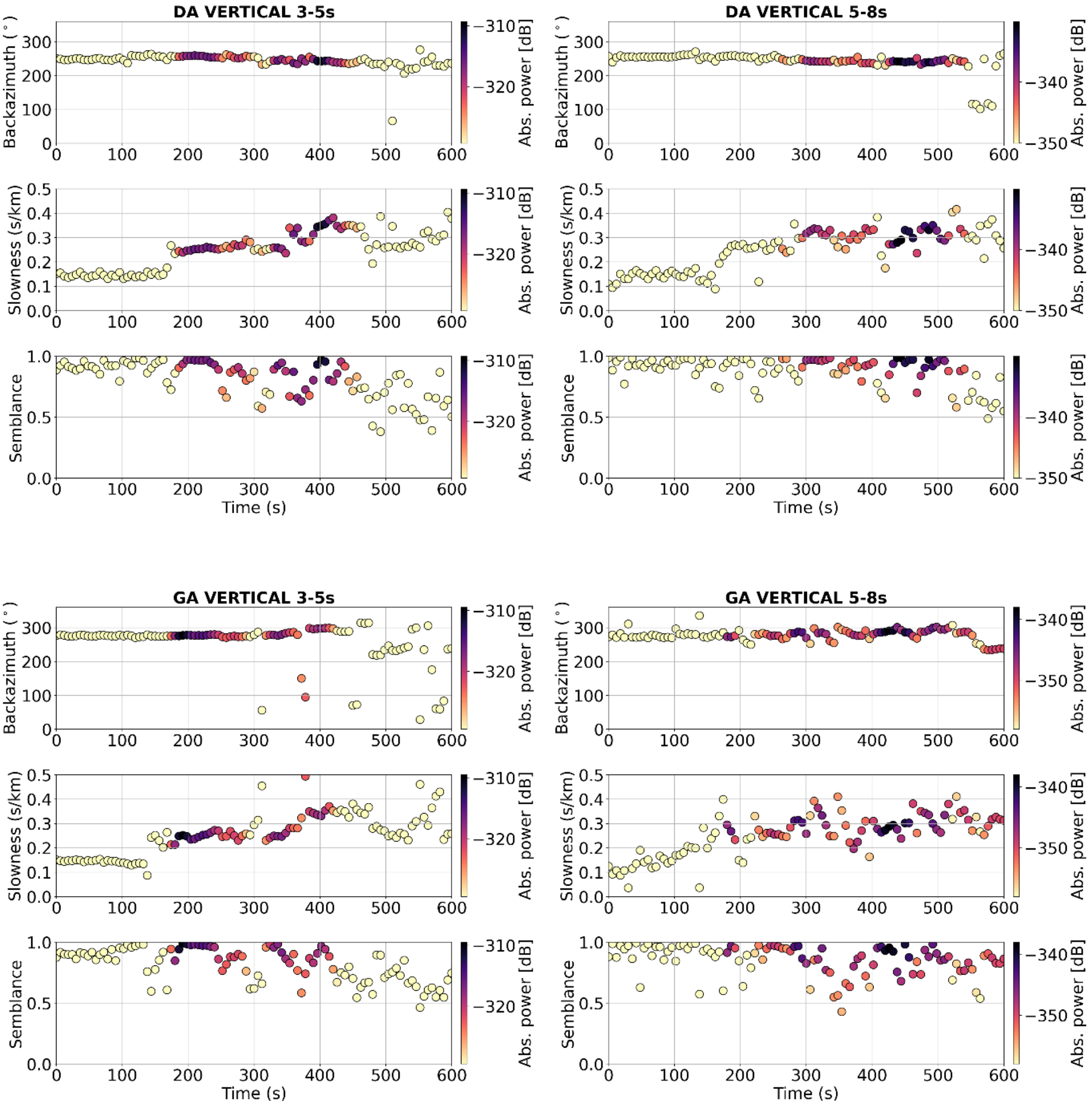

Supplementary Figure 5 - Array analysis for synthetic point source simulation. Back azimuth, slowness and semblance associated with vertical (Z) components of both Donegal (DA) and Galley Head (GA) arrays. For each array, the analysis is performed over a year of data in period bands 3-5s and 5-8s.

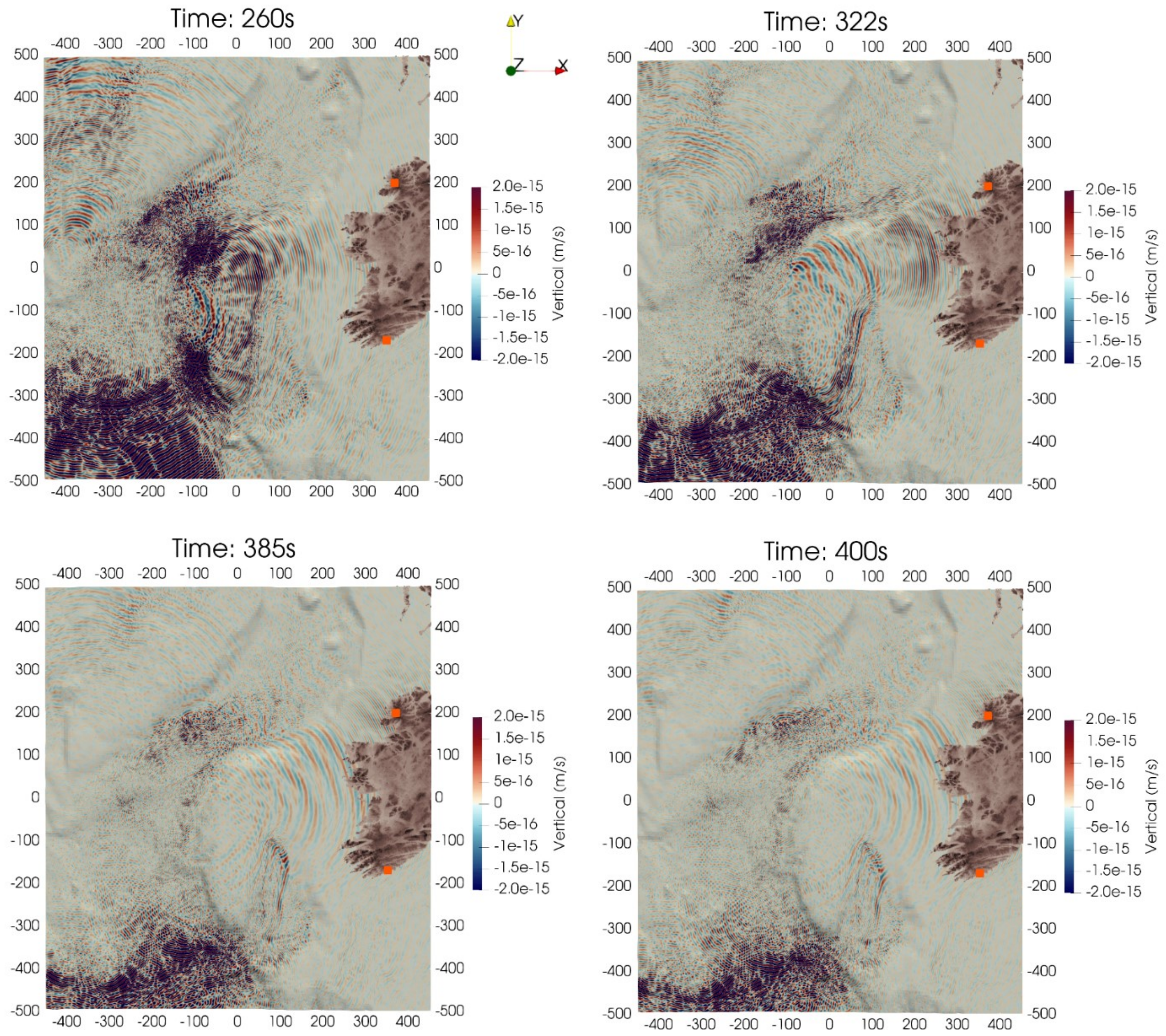

Supplementary Figure 6 - Snapshots for 3D path effects simulation. Propagation of the vertical wavefield for the simulation presented in Figure 3. The associated animation can be found in Supplementary Movie 1. It clearly shows how the propagating seismic wavefield is strongly affected by the margin morphology for a single acoustic point source located 15m below the sea surface, in the deep water. Each snapshot represents the projection of the wavefield 1km below the bathymetry.

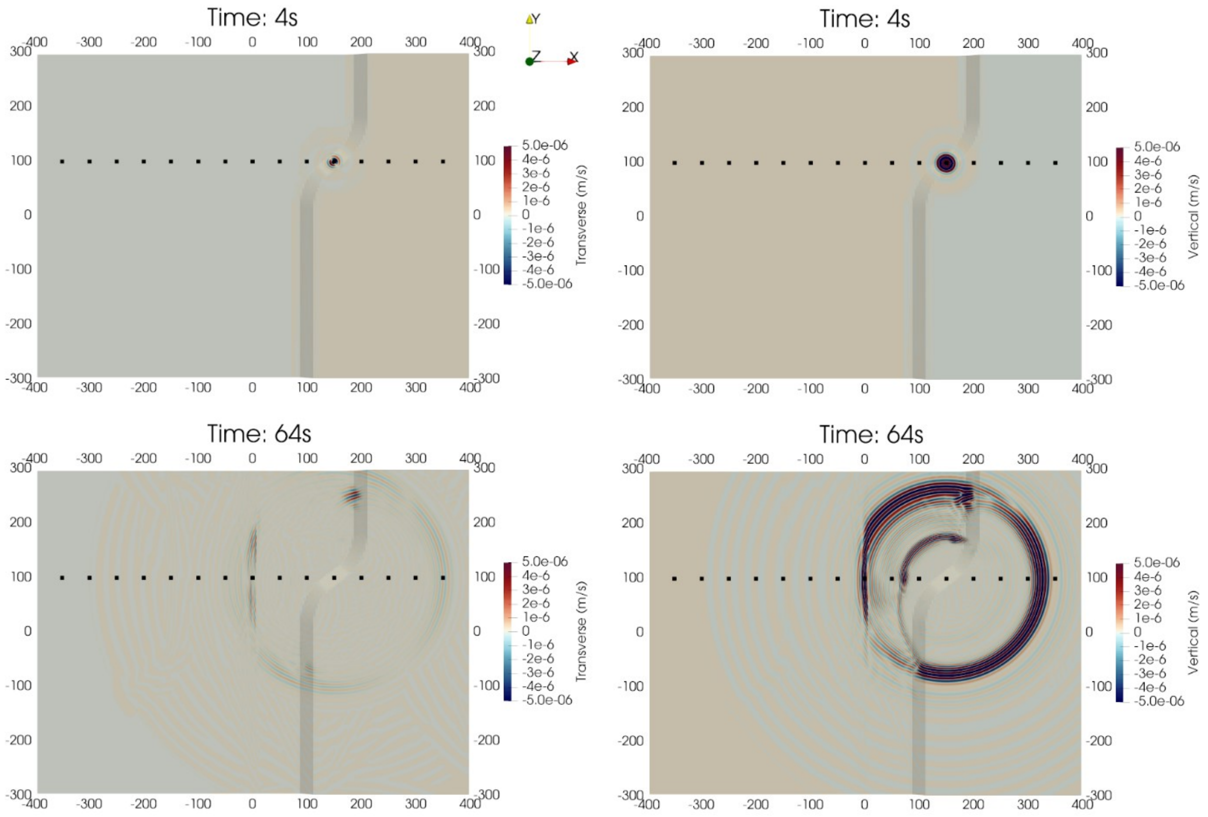

Supplementary Figure 7 - Snapshots for the source model S1 simulation. Both transverse (left panel) and vertical (right panel) wavefields are shown as well as the location of profile P1. The associated animations can be found in Supplementary Movie 2 and 3 for the transverse and vertical wavefield respectively. Each snapshot represents the projection of the wavefield 1km below the bathymetry.

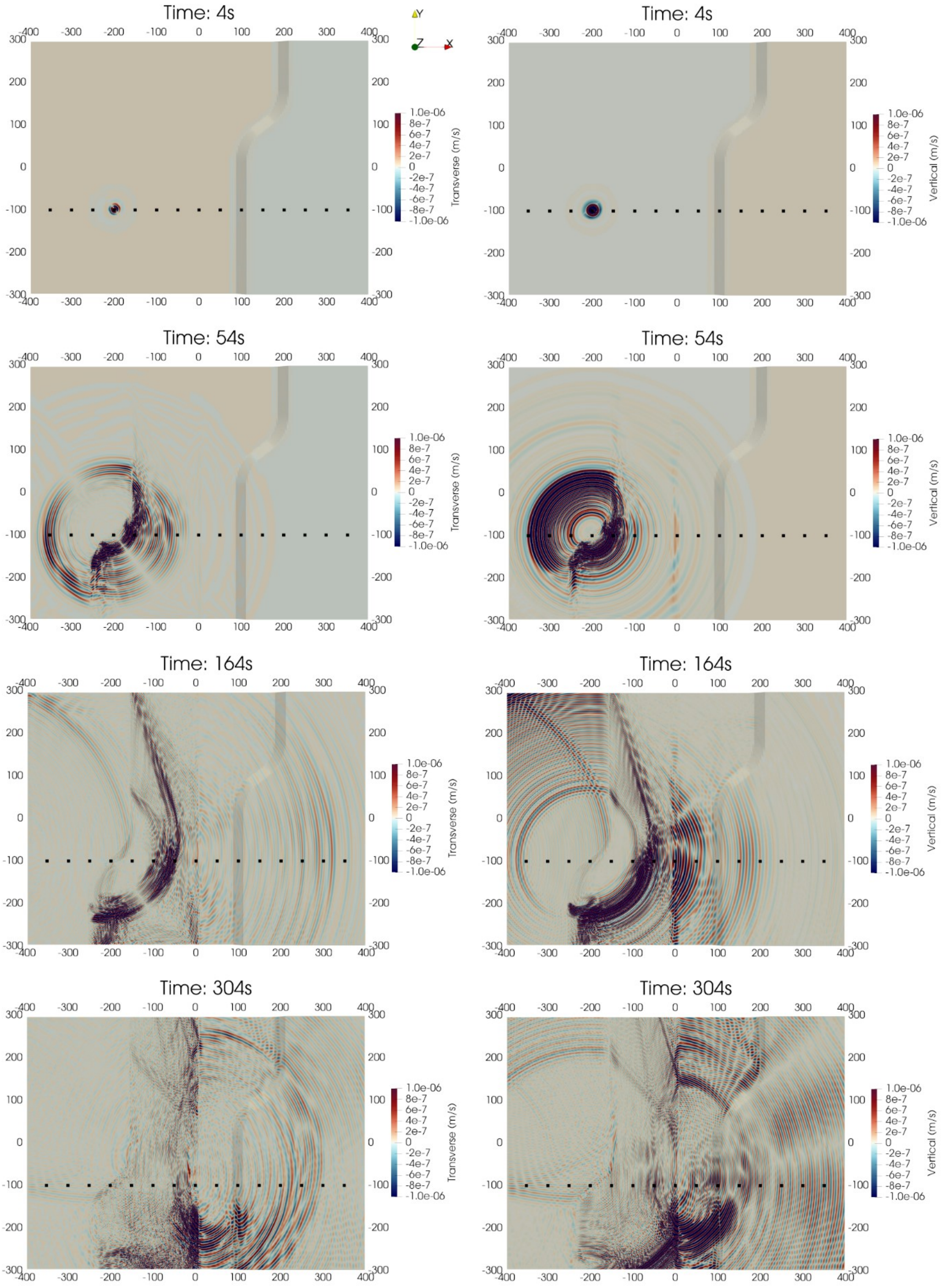

Supplementary Figure 8 - Snapshots for the source model S2 simulation. Both transverse (left panel) and vertical (right panel) wavefields are shown as well as the location of profile P2.

The associated animations can be found in Supplementary Movie 4 and 5 for the transverse and vertical wavefield respectively. Each snapshot represents the projection of the wavefield 1km below the bathymetry. It is worth noting the side reflection from the edge of the model (left side). This artefact appears strong due to the colour scale used; however it shows negligible effects on the synthetic data of interest as seen on Figure 4c.

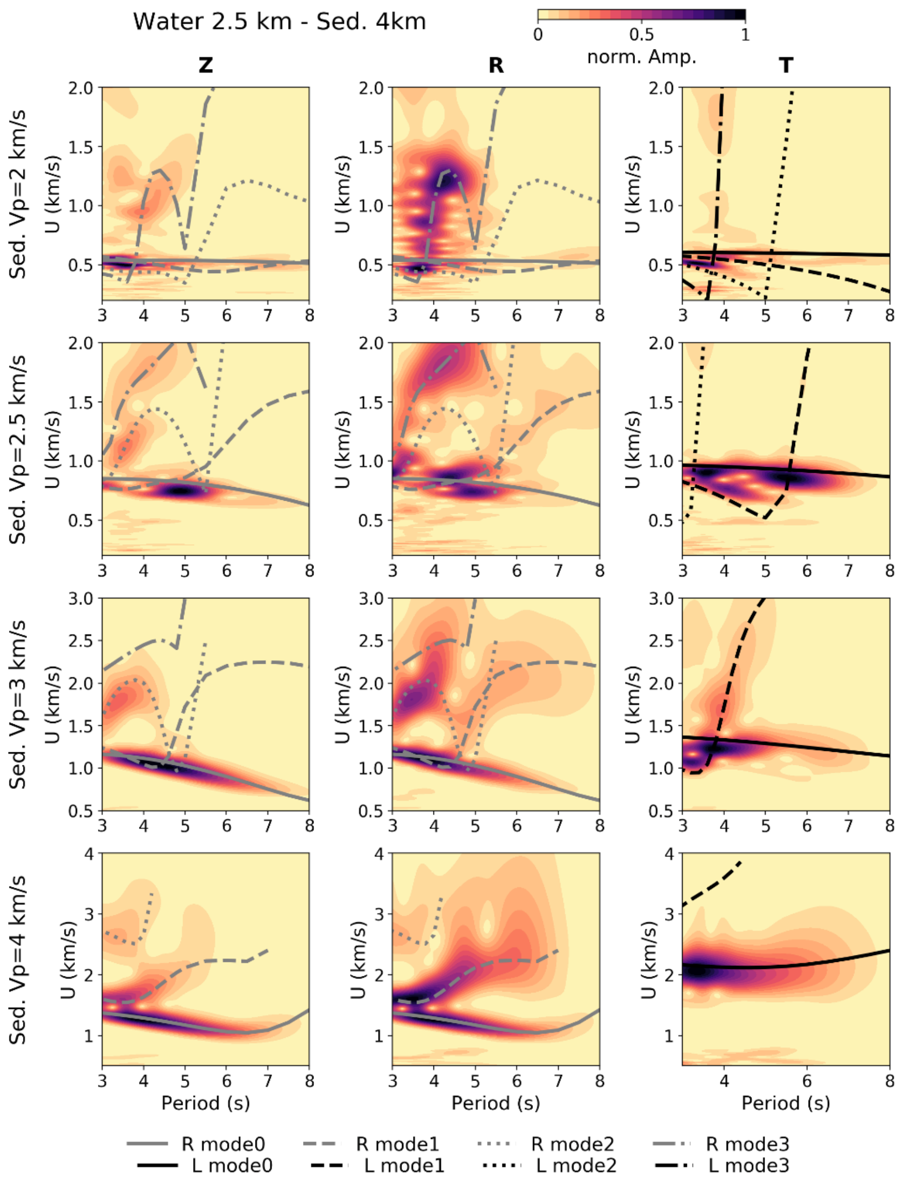

Supplementary Figure 9 - Dispersion analysis for Water 2.5 km deep and Sediments 4 km thick 3D model. The dispersion analysis is performed for the source configuration of source model 2 (Fig. 4c). The data are extracted on the sedimentary basin at station st07 (Fig. 4c). On each plot the amplitude is normalized over all periods in order to see phases of higher energy. Group velocity (U) against period is shown for all three components: vertical (Z), radial (R) and transverse (T), and for varying sediment velocities. Theoretical dispersion curves for the associated 1D model at station st07 are computed using the "computer programs in seismology" software package[1]. They are shown for the first four modes of Rayleigh (grey lines) and Love waves (black lines).

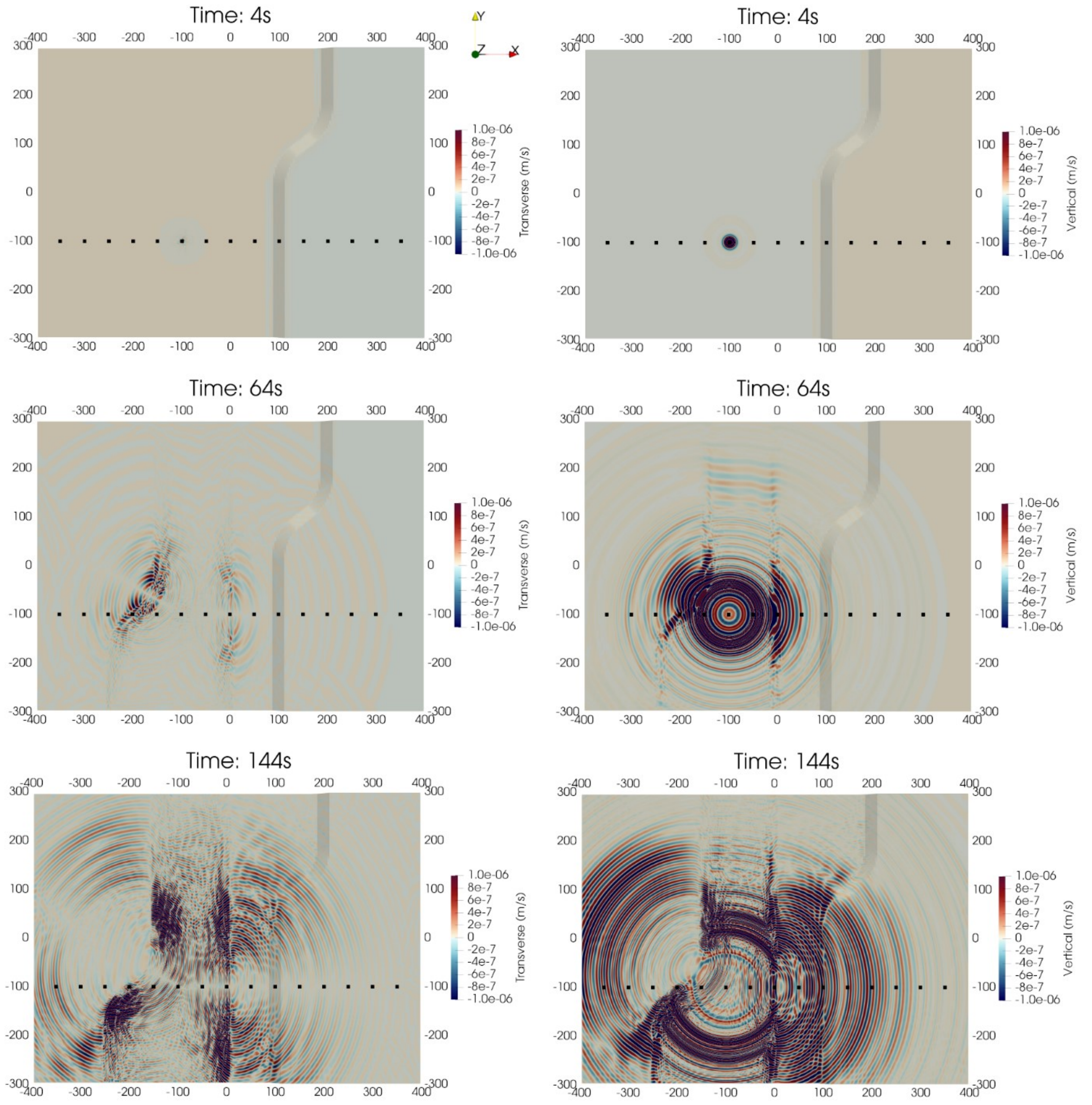

Supplementary Figure 10 - Snapshots for the source model S3 simulation. Both transverse (left panel) and vertical (right panel) wavefields are shown as well as the location of profile P2. The associated animations can be found in Supplementary Movie 6 and 7 for the transverse and vertical wavefield respectively. Each snapshot represents the projection of the wavefield 1km below the bathymetry.

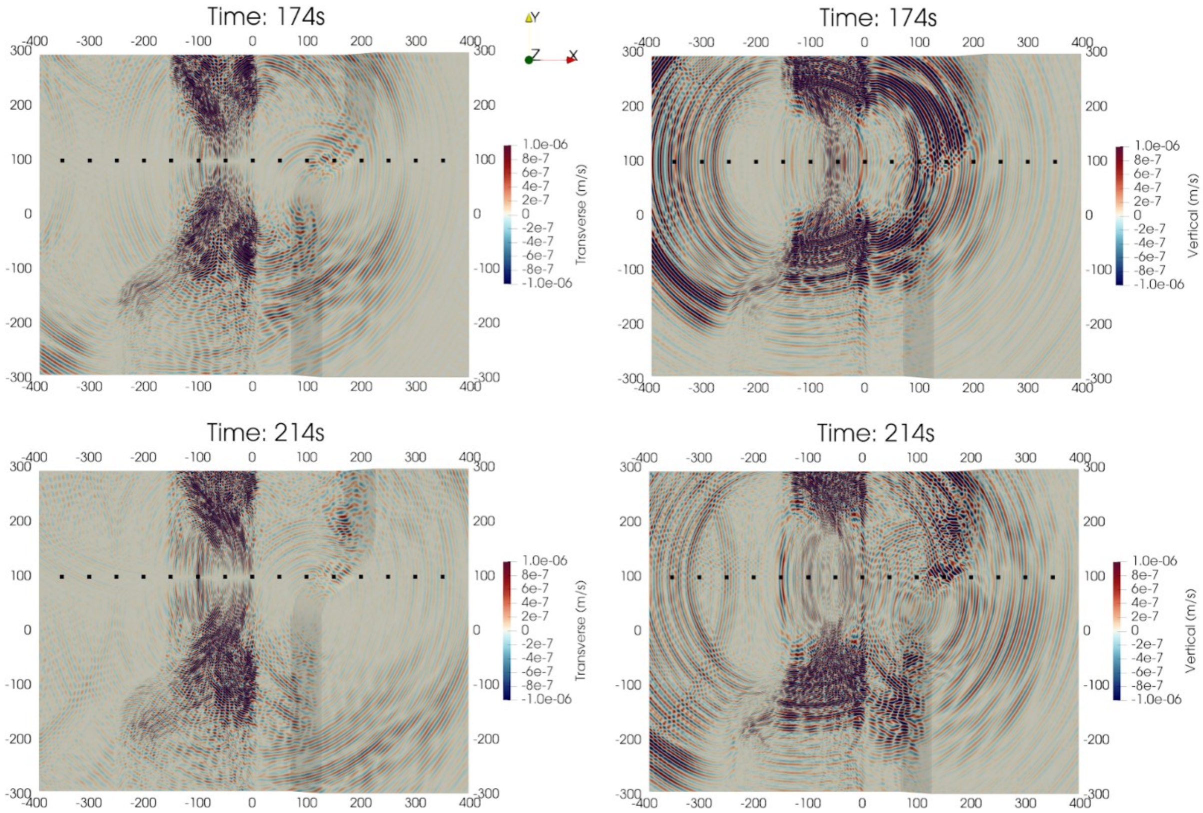

Supplementary Figure 11 - Snapshots for the source model S4 simulation. Both transverse (left panel) and vertical (right panel) wavefields are shown as well as the location of profile P1. The associated animations can be found in Supplementary Movie 8 and 9 for the transverse and vertical wavefield respectively. Each snapshot represents the projection of the wavefield 1km below the bathymetry.

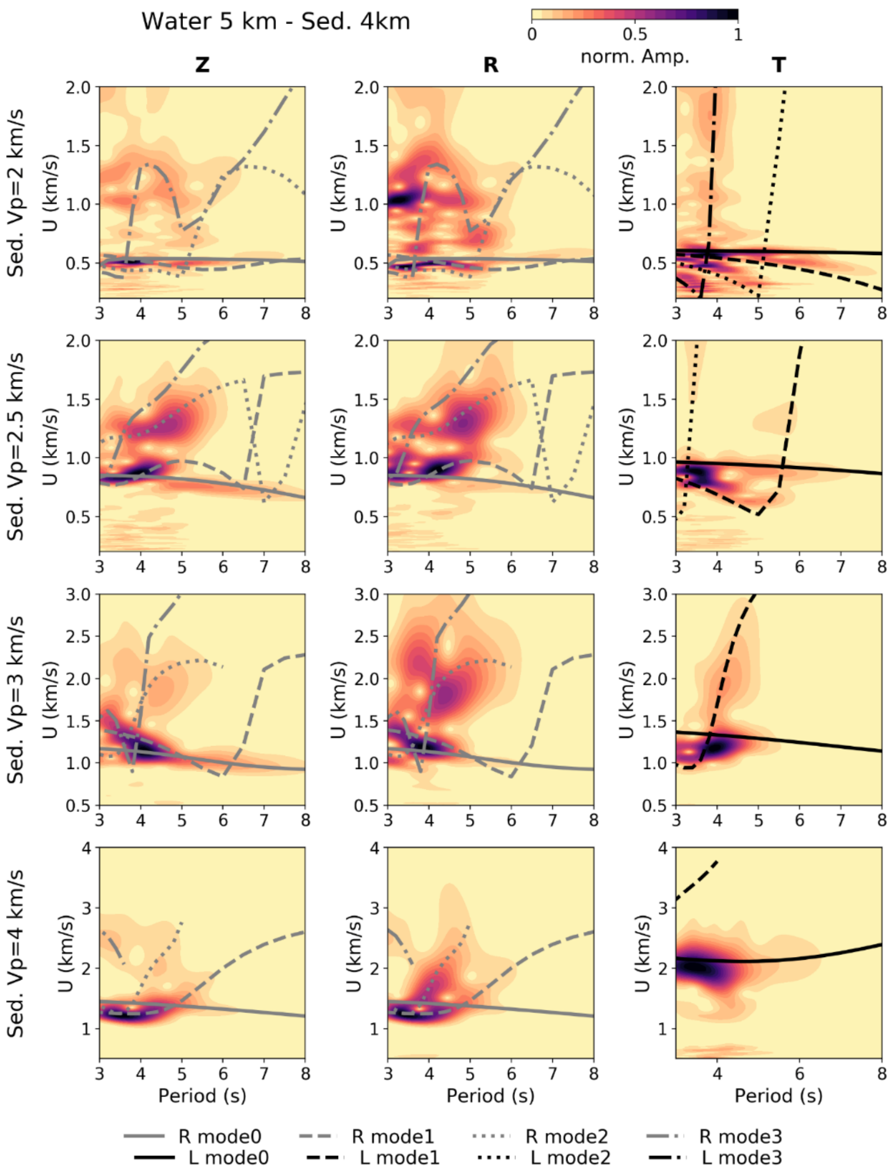

Supplementary Figure 12 - Dispersion analysis for Water 5 km deep and Sediments 4 km thick 3D model. The dispersion analysis is performed for the source configuration of source model 2 (Fig. 4c). The data are extracted on the sedimentary basin at station st07 (Fig. 4c). On each plot the amplitude is normalized over all periods in order to see phases of higher energy. Group velocity (U) against period is shown for all three components: vertical (Z), radial (R) and transverse (T), and for varying sediment velocities. Theoretical dispersion curves for the associated 1D model at station st07 are computed using the "computer programs in seismology" software package[1]. They are shown for the first four modes of Rayleigh (grey lines) and Love waves (black lines).

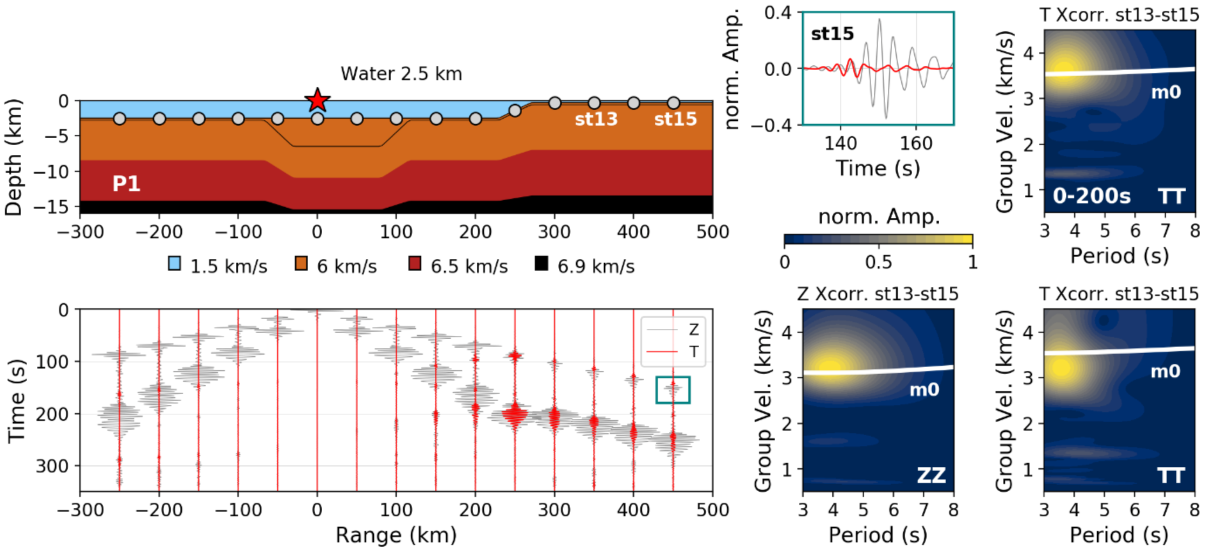

Supplementary Figure 13 - Rayleigh to Love wave conversions. Source model S4 with no sediments and water depth of 2.5 km: source located above the position of the sedimentary basin (when present). Cross-section of the 3D model along profile P1 as well as Z and T components normalized (based on vertical amplitude) seismograms for all stations along the profile are shown. In this configuration, the profile P1 is perpendicular to the sedimentary basin geometry but crosses a change in the bathymetry lateral geometry (Fig. 4a) enabling observation of Rayleigh to Love wave conversions at the shelf area for the pseudo-Rayleigh wave first overtone. However, the pseudo-Rayleigh fundamental mode interaction with the shelf leads to quasi-Rayleigh waves on the transverse component. Due to the inline configuration of source and receivers, dispersion analysis is performed for Z and T components through cross-correlation (Xcorr.) of pairs of stations in order to characterize the seismic wavefield on the shelf (st13-st15 pair). For comparison, theoretical dispersion curves for fundamental (m0) and first overtone (m1) for Rayleigh waves (ZZ) and Love waves (TT) associated with the 1D structure below each station pair are also displayed.

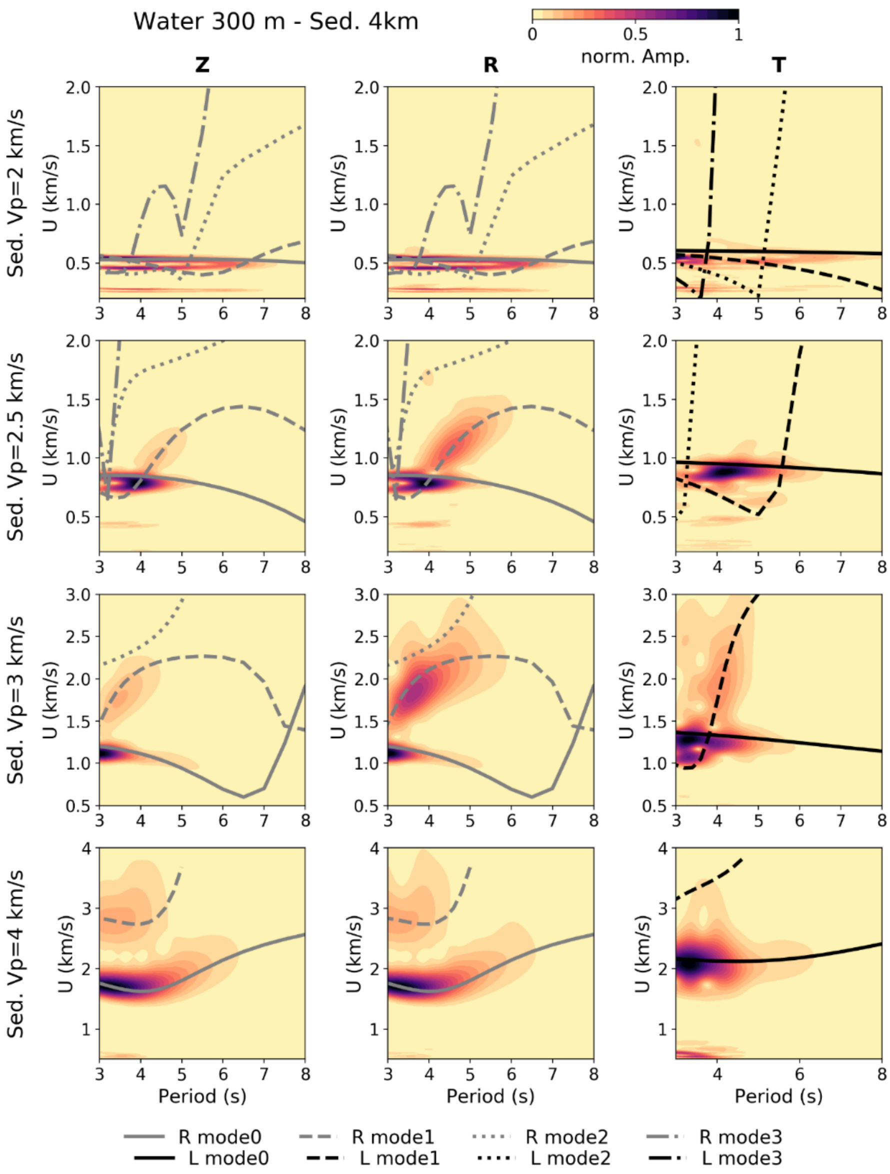

Supplementary Figure 14 - Dispersion analysis for Water 300 m deep and Sediments 4 km thick 3D model. The dispersion analysis is performed for the source configuration of source model 2 (Fig. 4c). The data are extracted on the sedimentary basin at station st07 (Fig. 4c). On each plot the amplitude is normalized over all periods in order to see phases of higher energy. Group velocity (U) against period is shown for all three components: vertical (Z), radial (R) and transverse (T), and for varying sediment velocities. Theoretical dispersion curves for the associated 1D model at station st07 are computed using the “computer programs in seismology” software package[1]. They are shown for the first four modes of Rayleigh (grey lines) and Love waves (black lines).

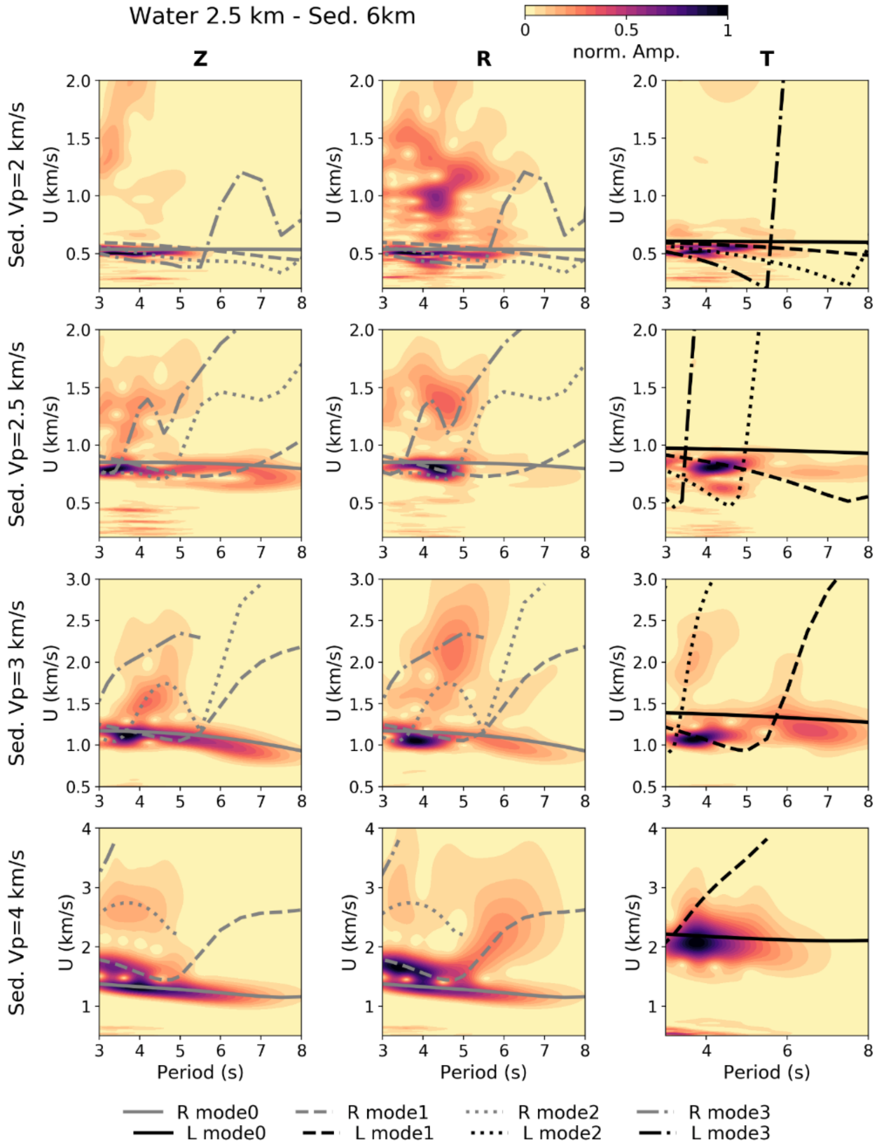

Supplementary Figure 15 - Dispersion analysis for Water 2.5 km deep and Sediments 6 km thick 3D model. The dispersion analysis is performed for the source configuration of source model 2 (Fig. 4c). The data are extracted on the sedimentary basin at station st07 (Fig. 4c). On each plot the amplitude is normalized over all periods in order to see phases of higher energy. Group velocity (U) against period is shown for all three components: vertical (Z), radial (R) and transverse (T), and for varying sediment velocities. Theoretical dispersion curves for the associated 1D model at station st07 are computed using the "computer programs in seismology" software package[1]. They are shown for the first four modes of Rayleigh (grey lines) and Love waves (black lines).

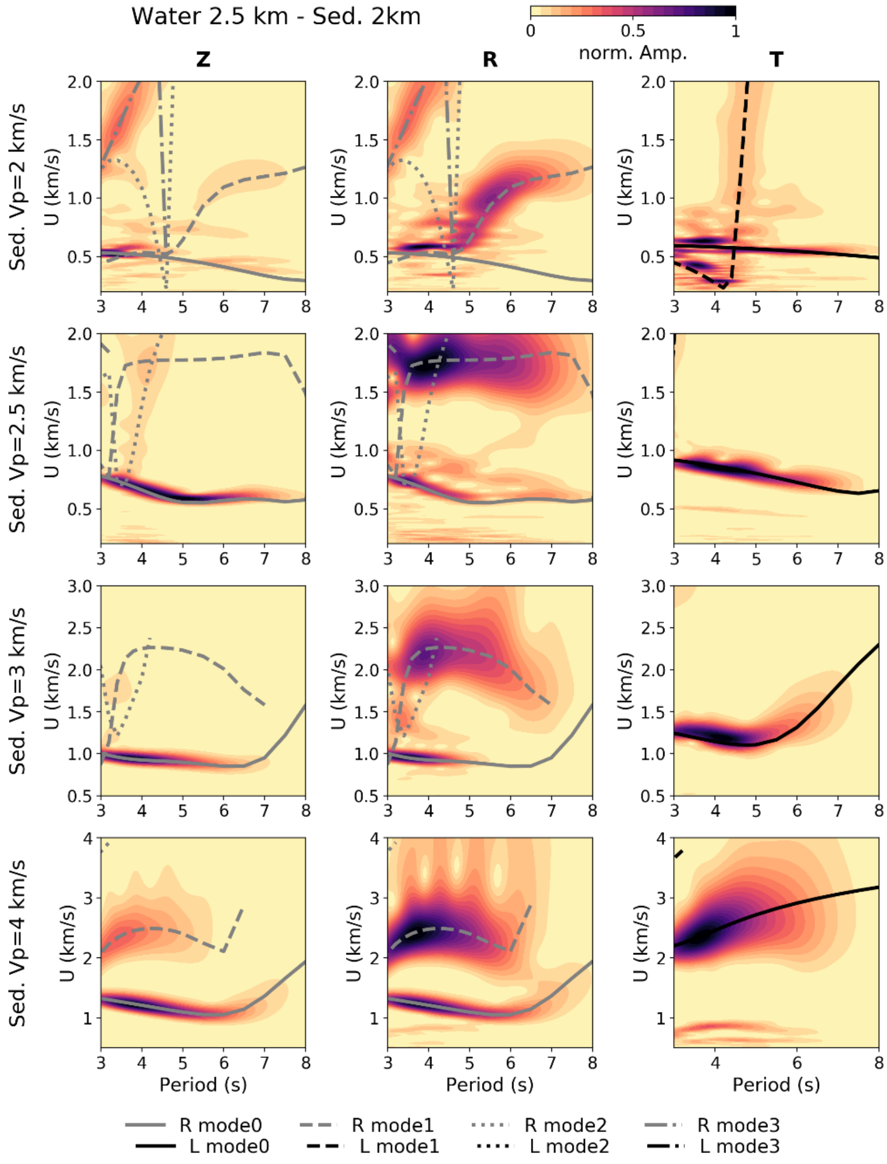

Supplementary Figure 16 - Dispersion analysis for Water 2.5 km deep and Sediments 2 km thick 3D model. The dispersion analysis is performed for the source configuration of source model 2 (Fig. 4c). The data are extracted on the sedimentary basin at station st07 (Fig. 4c). On each plot the amplitude is normalized over all periods in order to see phases of higher energy. Group velocity (U) against period is shown for all three components: vertical (Z), radial (R) and transverse (T), and for varying sediment velocities. Theoretical dispersion curves for the associated 1D model at station st07 are computed using the "computer programs in seismology" software package[1]. They are shown for the first four modes of Rayleigh (grey lines) and Love waves (black lines).

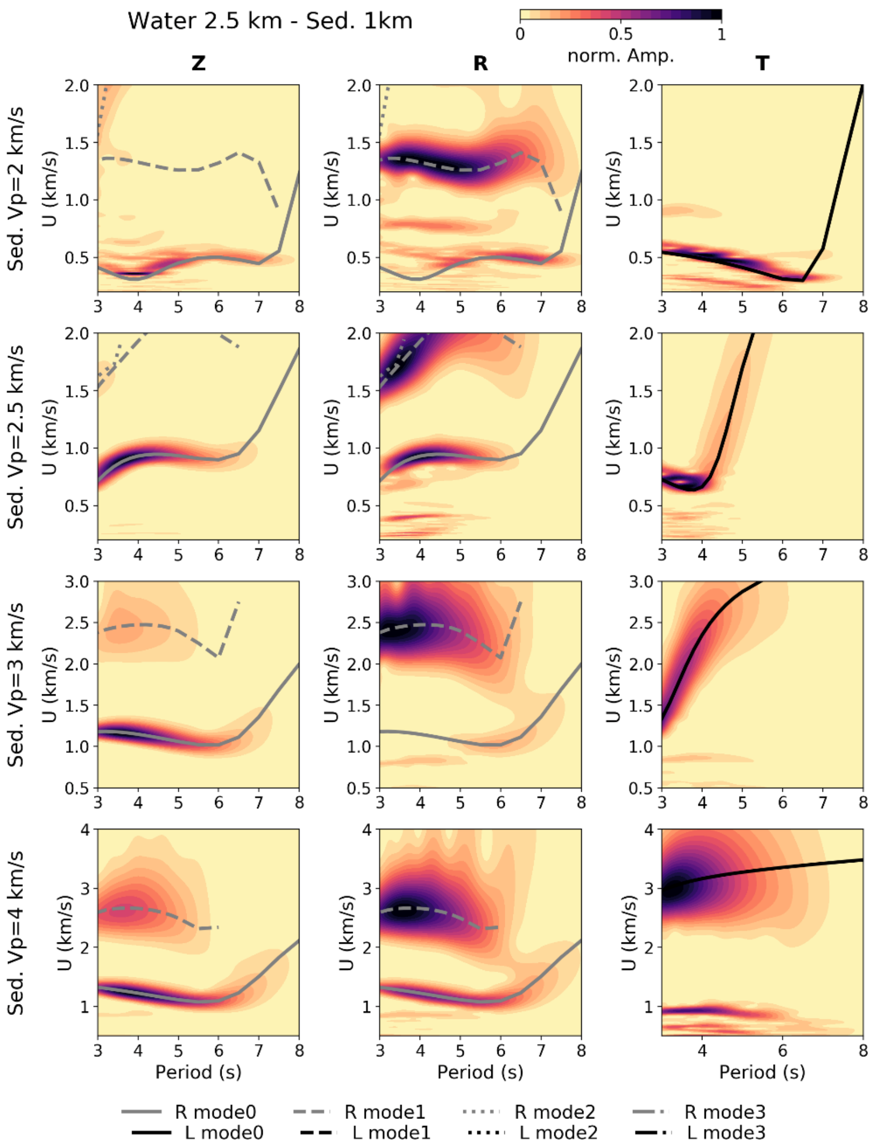

Supplementary Figure 17 - Dispersion analysis for Water 2.5 km deep and Sediments 1 km thick 3D model. The dispersion analysis is performed for the source configuration of source model 2 (Fig. 4c). The data are extracted on the sedimentary basin at station st07 (Fig. 4c). On each plot the amplitude is normalized over all periods in order to see phases of higher energy. Group velocity (U) against period is shown for all three components: vertical (Z), radial (R) and transverse (T), and for varying sediment velocities. Theoretical dispersion curves for the associated 1D model at station st07 are computed using the "computer programs in seismology" software package[1]. They are shown for the first four modes of Rayleigh (grey lines) and Love waves (black lines).

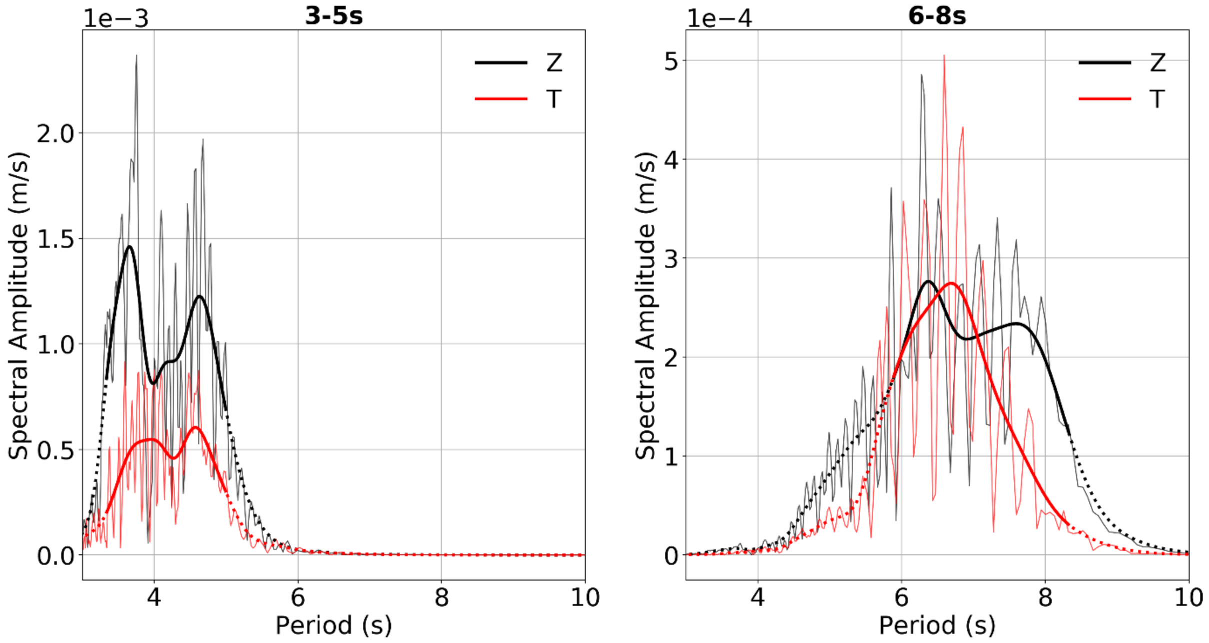

Supplementary Figure 18 - L/R ratios calculation method. The L/R kinetic energy ratios are estimated based on the spectral amplitude ratio T/Z between transverse and vertical components (see method section). Here, the T/Z ratio is estimated as the mean ratio between smoothed transverse and vertical spectrum for two period bands 3-5s and 6-8s. This leads to a T/Z ratio estimate centred on periods 4s and 7s: the periods of eigenfunctions used for the L/R calculations (Supp. Fig. S19). The spectrum smoothing filter is based on the Konno-Ohmachi method[2] and implemented in Obspy[3]. Data shown is taken from synthetic station st15 for source model 2 (Fig. 4c).

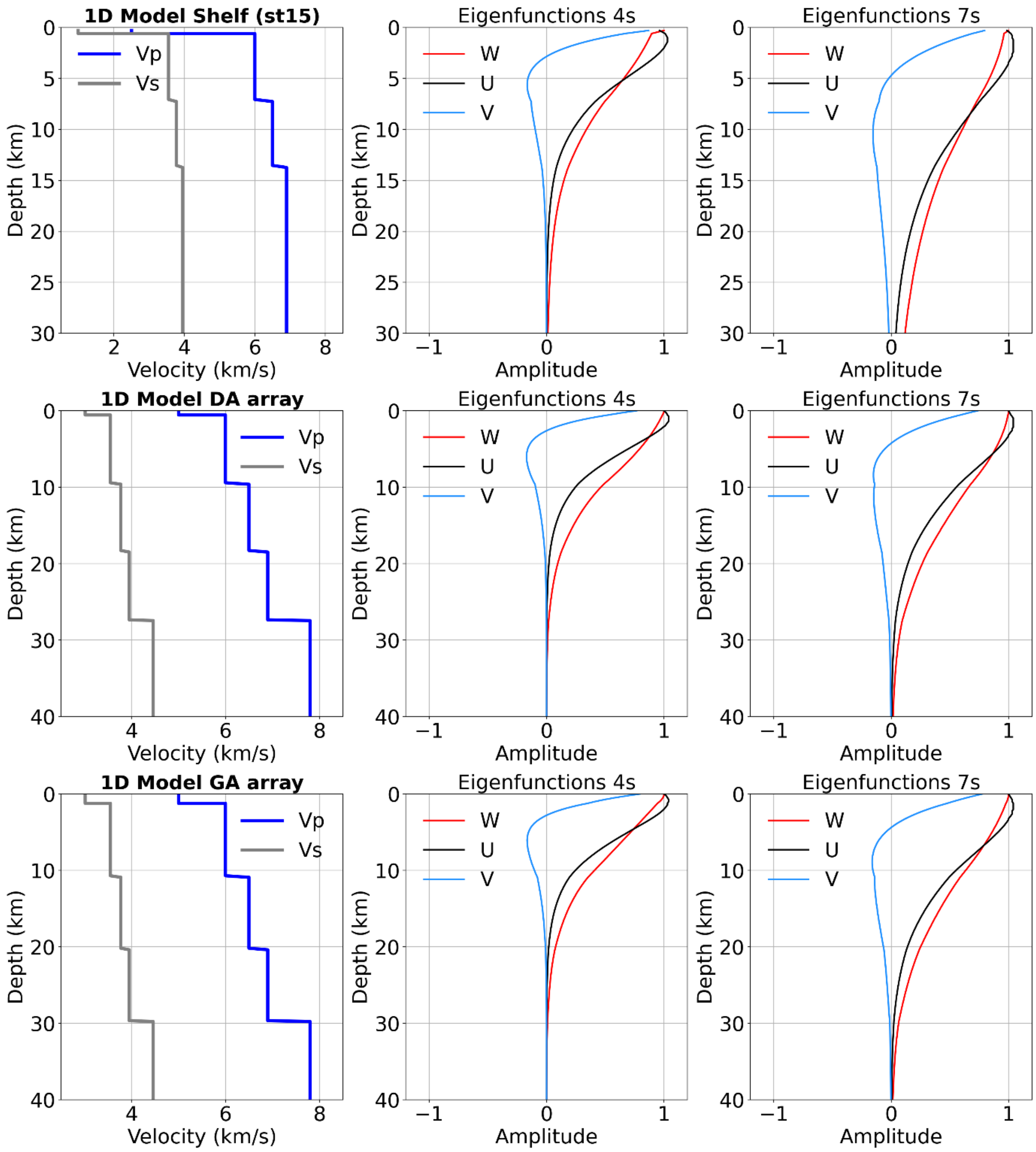

Supplementary Figure 19 - Rayleigh and Love waves eigenfunctions. The fundamental mode eigenfunctions are derived from different 1D models for st15 from the 3D concept model (Fig. 4), DA and GA arrays and are used to calculate Love to Rayleigh wave energy ratios (see method section). They are represented at characteristic secondary microseism periods (4s and 7s) for Love wave (W), Rayleigh vertical (U) and Rayleigh radial (V) components.

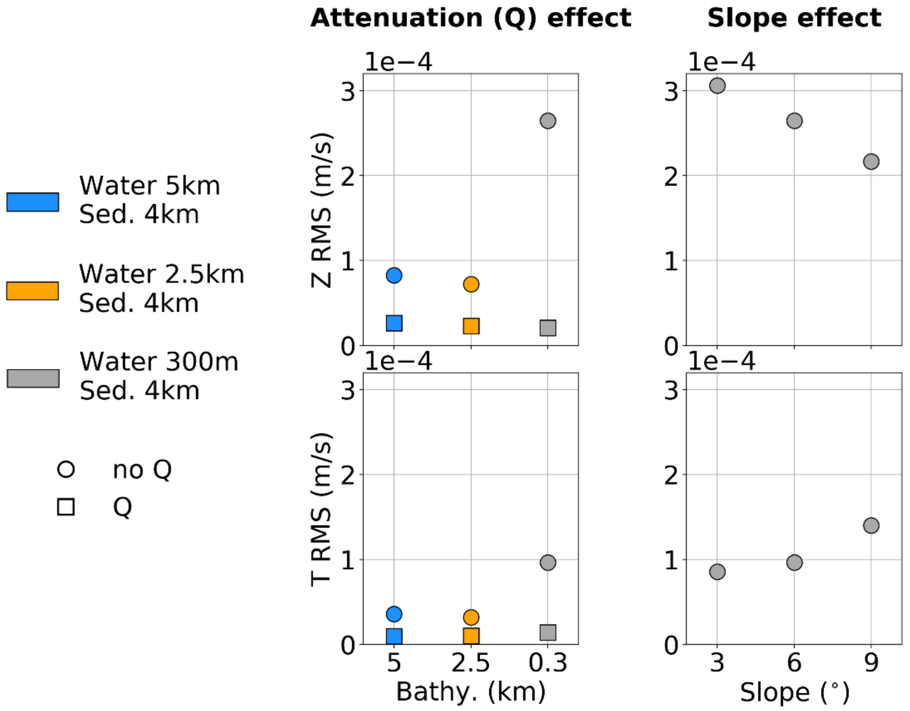

Supplementary Figure 20 - Attenuation (Q) and slope effects on L/R ratios. In complement to Figure 6, here is presented the effect of including attenuation in the simulations and changing the slope of the sedimentary basin for simulations associated with source model S2. The RMS amplitude of both vertical (Z) and transverse (T) components (3-8s) are associated with station st15 on profile P2 (R2). Here, the sediment velocity used in all the simulations is 2.5 km/s.

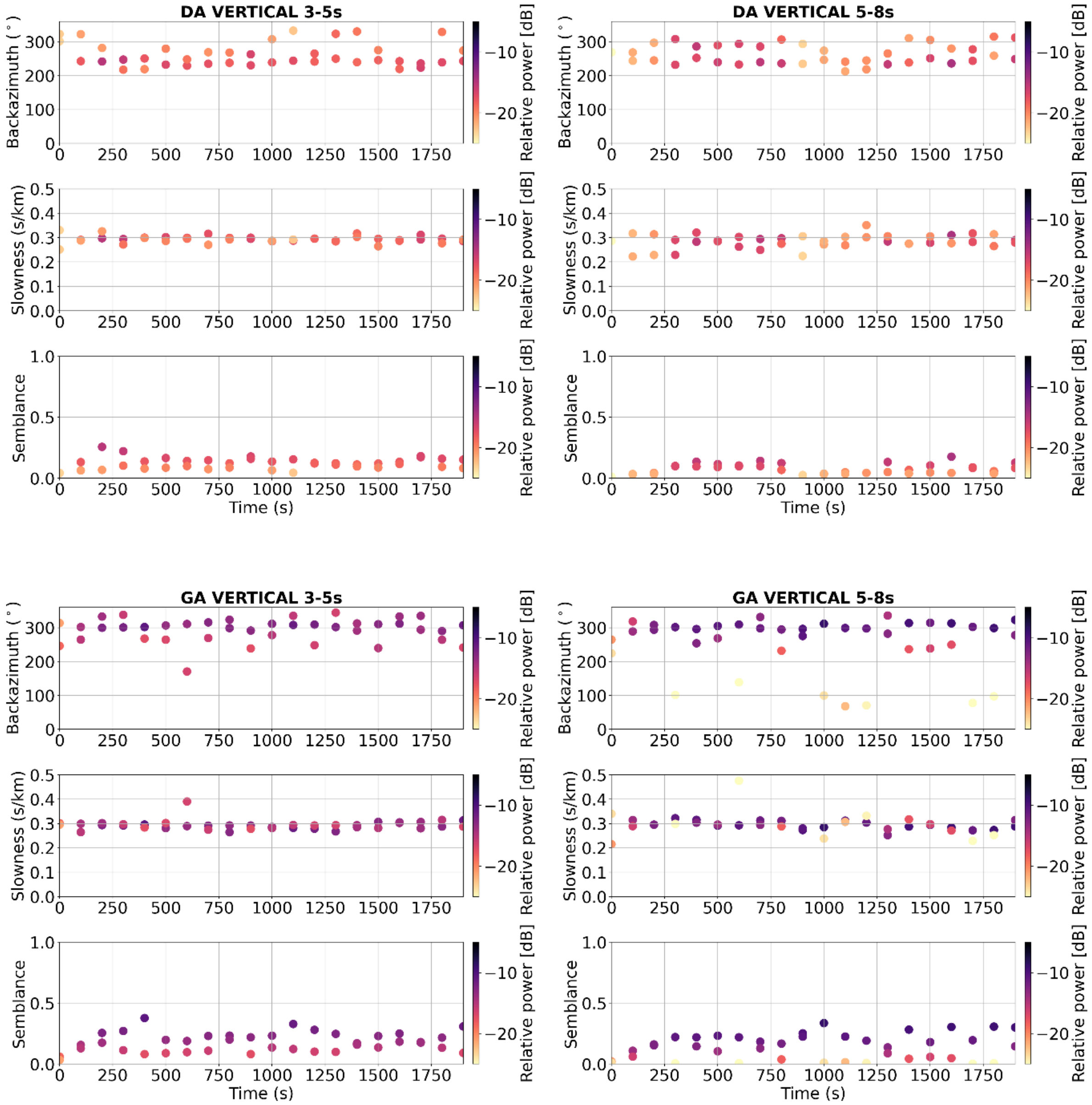

Supplementary Figure 21 - Array analysis for synthetic P2L source simulation (Z component). Back azimuth, slowness and semblance associated with vertical (Z) components of both Donegal (DA) and Galley Head (GA) arrays. For each array, the analysis is performed for period bands 3-5s and 5-8s.

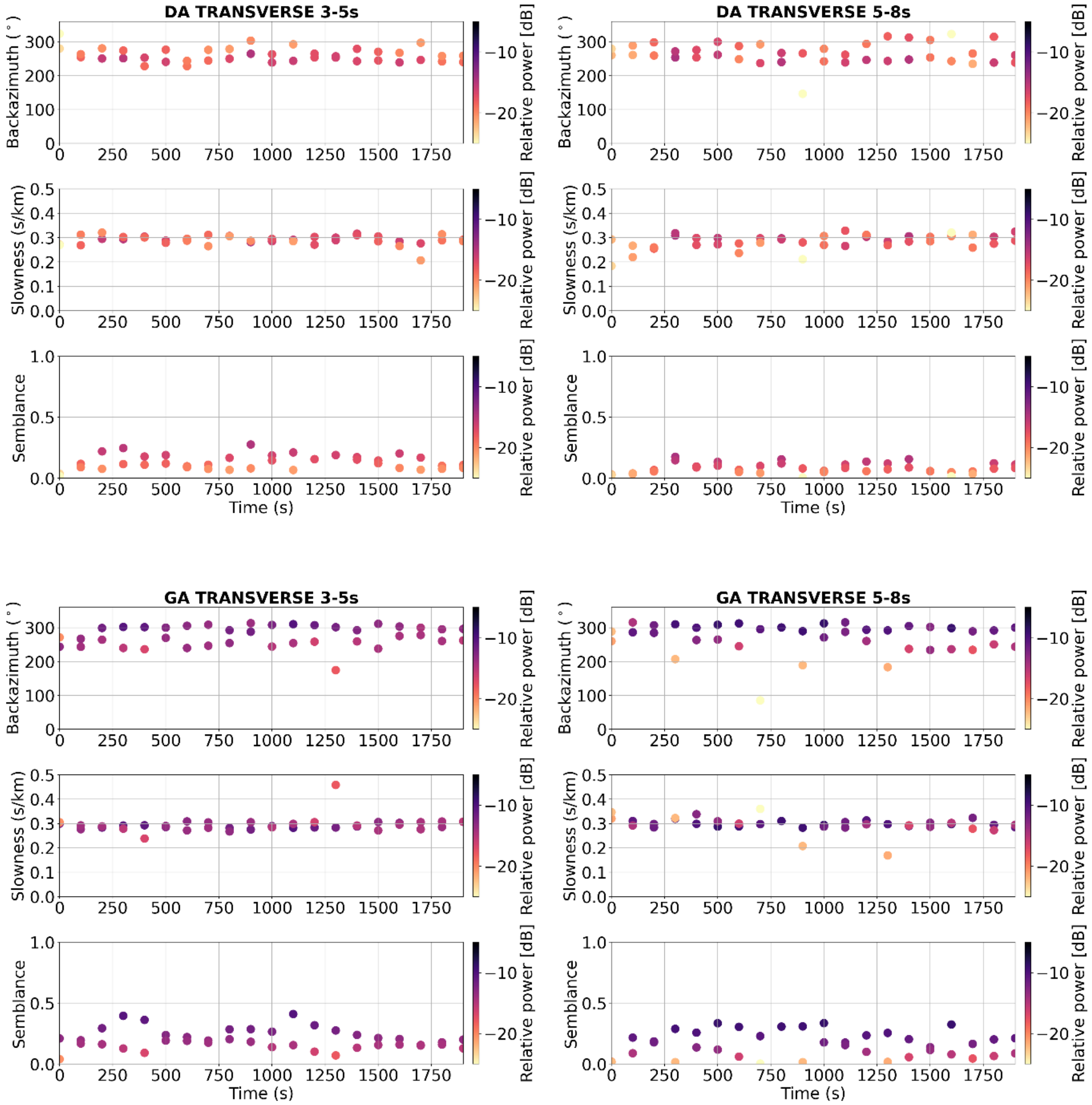

Supplementary Figure 22 - Array analysis for synthetic P2L source simulation (T component). Back azimuth, slowness and semblance associated with transverse (T) components of both Donegal (DA) and Galley Head (GA) arrays. For each array, the analysis is performed for period bands 3-5s and 5-8s.

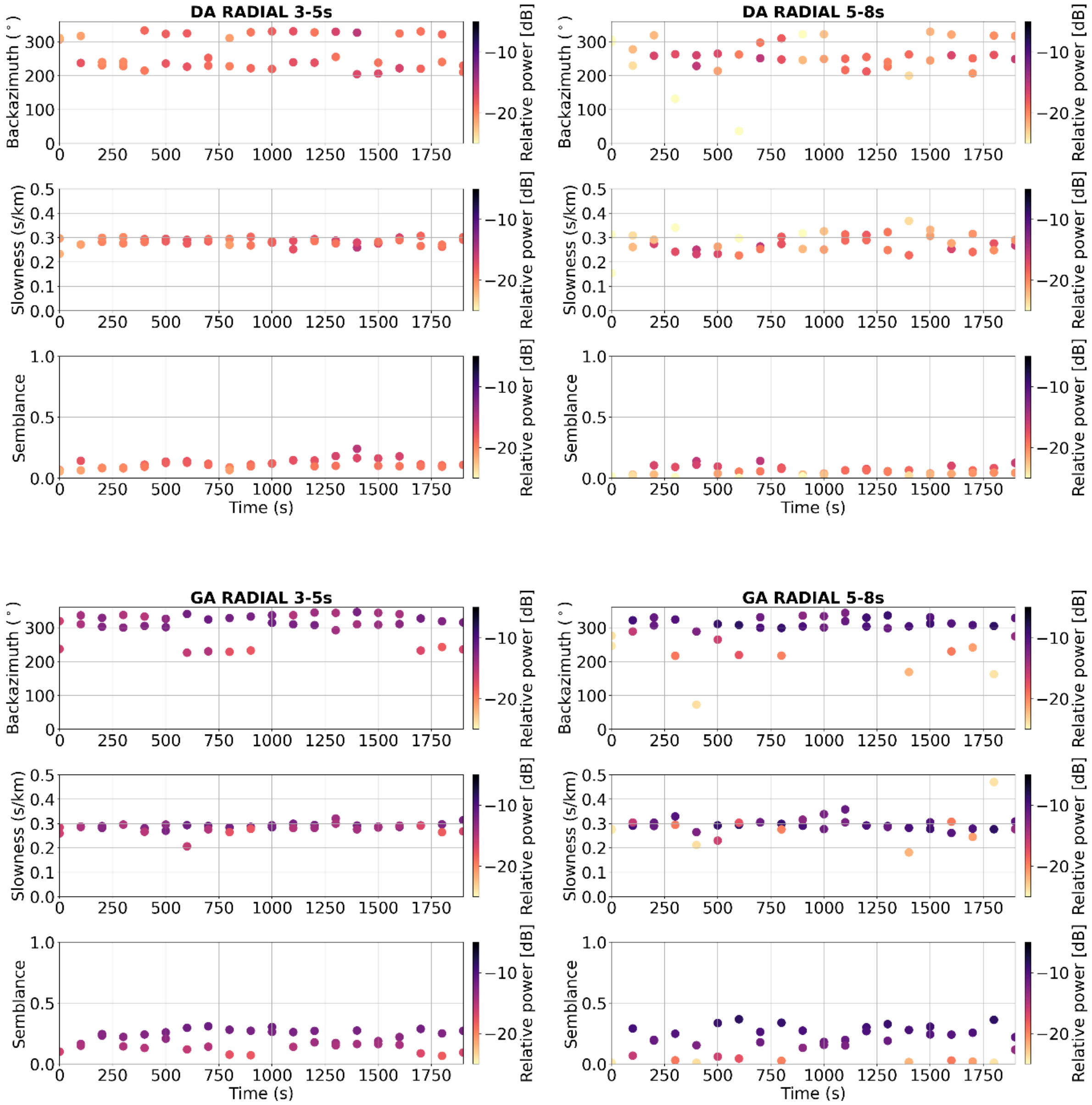

Supplementary Figure 23 - Array analysis for synthetic P2L source simulation (R component). Back azimuth, slowness and semblance associated with radial (R) components of both Donegal (DA) and Galley Head (GA) arrays. For each array, the analysis is performed for period bands 3-5s and 5-8s.

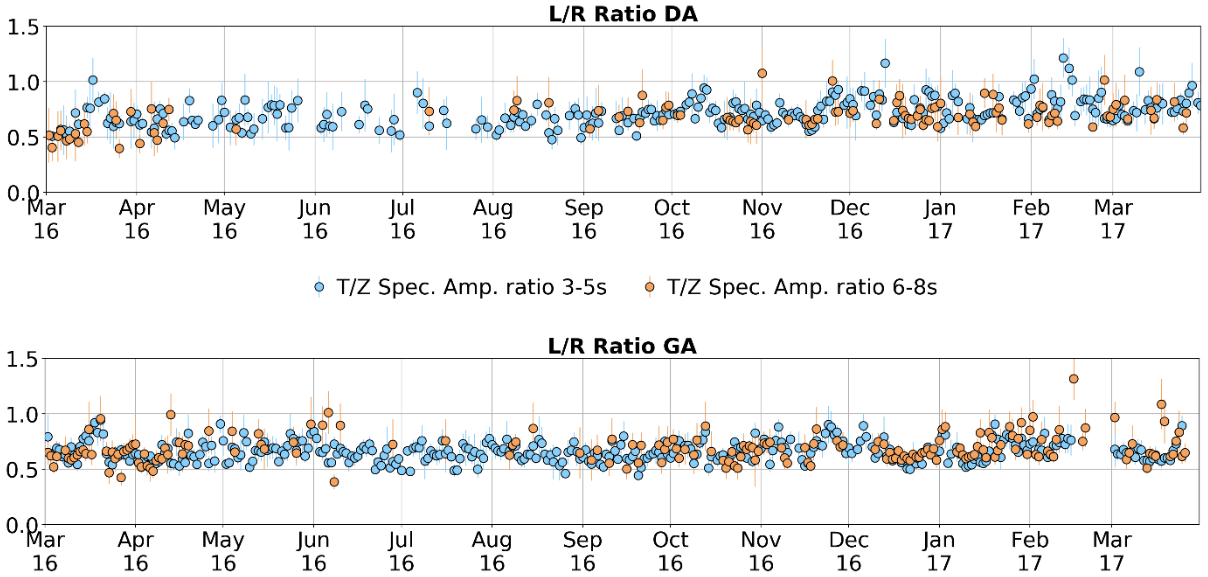

Supplementary Figure 24 – Daily L/R ratios in Ireland. Observed transverse over vertical components (T/Z) spectral amplitude ratios for both DA and GA arrays and period band 3-5s (blue) and 6-8s (orange). Each daily T/Z ratio is estimated from the average T/Z ratios over the array processing time windows for each day. Only the data points with standard deviations below 0.25 are plotted to see more clearly the robust estimates, meaning stable T/Z estimates through the day. The gap observed in the summer months particularly for the 6-8s period bands likely reflect the presence of more diffuse secondary microseisms sources.

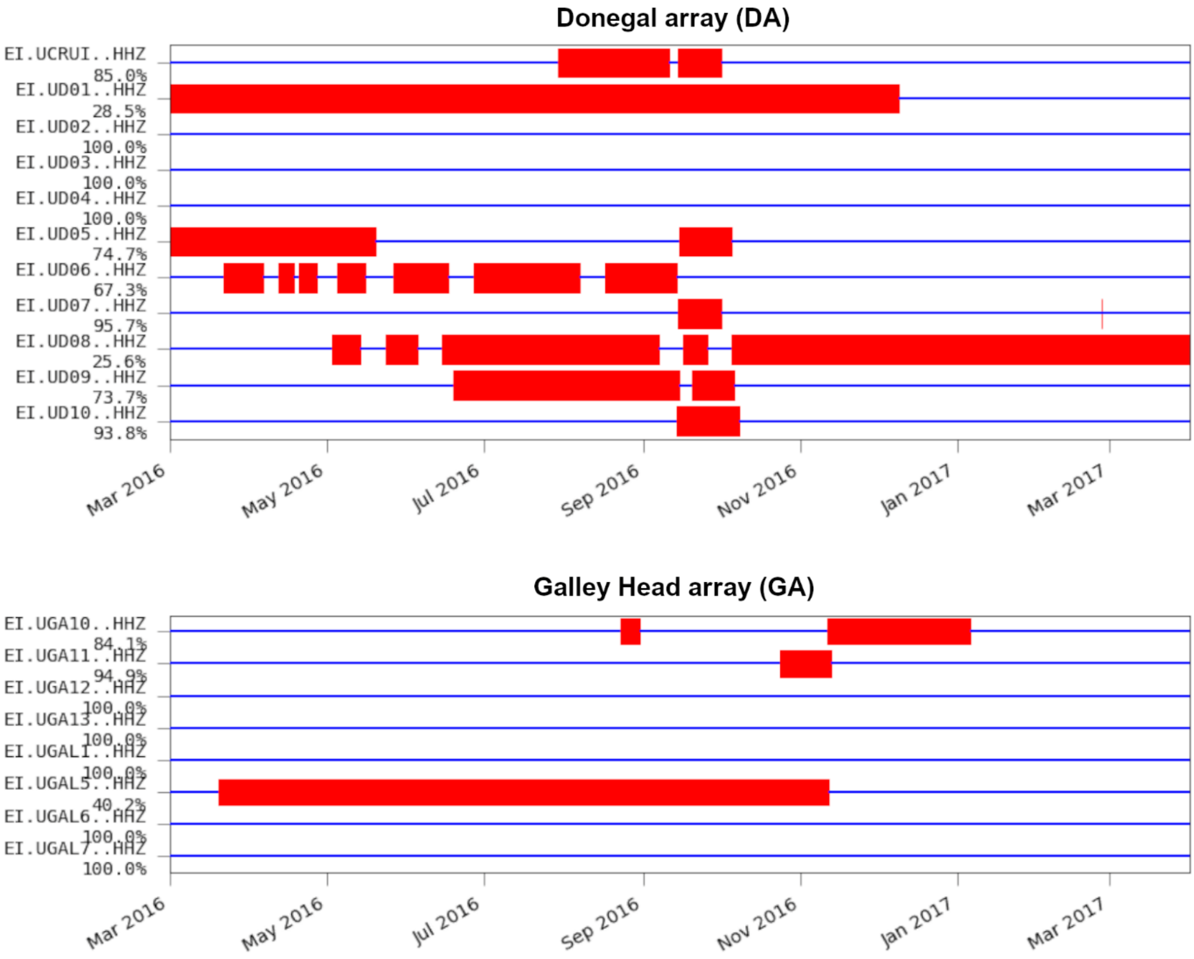

Supplementary Figure 25 – Data availability at the Field Arrays. The plots highlight the available data (thin blue horizontal line) and gaps (thick red bands) for the full period of the array analysis performed on the real data.

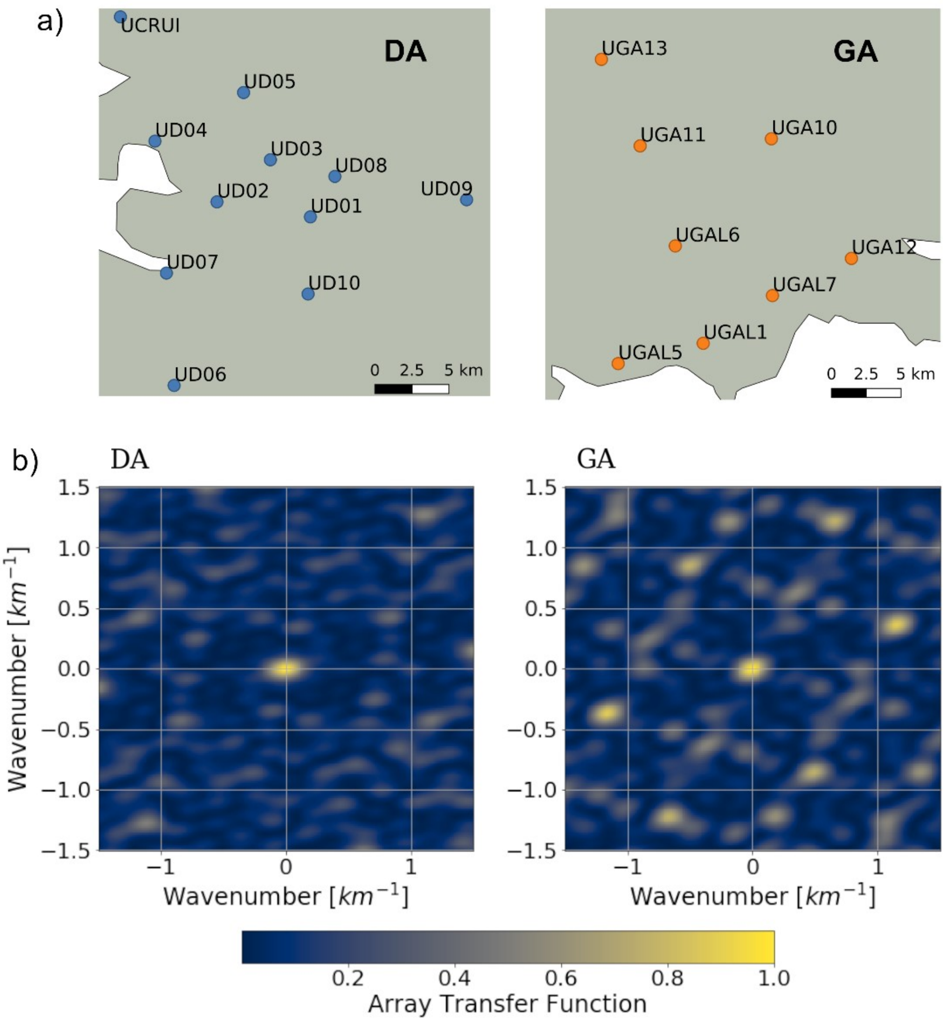

Supplementary Figure 26 – DA and GA arrays configuration. a) Arrays geometry; **b)** Arrays response function[4]: Secondary microseisms (SM) typically occur between 3 and 10 s, which corresponds to wavenumbers between 0.2 and 0.6 km^{-1} (assuming a velocity of 3.3 km/s which is typical for the fundamental Rayleigh mode in the region at SM frequencies). The wavenumbers that the arrays are sensitive to, can be defined as the limits k_{min} and k_{max}. k_{min} is the width of the main lobe at the half-power level and approximates the resolution capabilities of the array (that is, its ability to distinguish signals with similar wavenumbers). k_{max} estimates the aliasing limits of the array. It describes the proximity of secondary lobes to the main lobe position (that is, where the closest secondary lobe reaches the half-power level)[5]. For DA the main lobe is almost Gaussian in appearance and there are no significant secondary lobes between 1 km^{-1} showing that DA is well suited for the analysis of SM. For GA, the main lobe

is also Gaussian-like with some elongation in the north-east to south-west direction. The closest secondary lobe occurs at ~1 km^{-1} showing this array is also suitable for SM surface wave analysis. For a single source, k_{max} does not effectively limit the performance of the array. However, in the case of multiple sources, the superposition of responses can lead to secondary lobes being confused with the main lobe. For this reason, we use Geopsy's high resolution Capon's method for the array analysis.

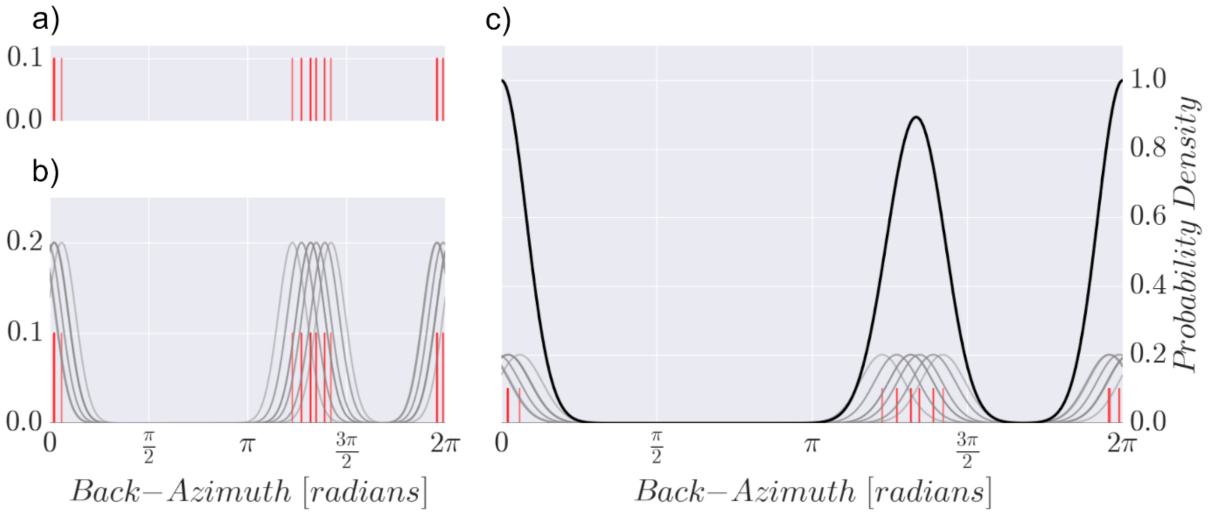

Supplementary Figure 27 – Probability density kernels estimation. a) Rug plot of back azimuths (BAZ). It is a 1-D representation of the density of back-azimuths recorded over a period of one hour at the DA array. The data is bi-modal as two clusters are observed near 3π/2 and 2π. The cluster near 2π is important as BAZ occur at either side of the zero point. Since BAZ are a circular quantity the resulting distribution needs to be wrapped from 0 to 2π. To achieve this, the von Mises distribution is used as the kernel. At each sample point the von Mises distribution is specified spreading the uncertainty in the measurement throughout the sample space (0 to 2π); **b)** Von Mises distribution centred at each data point in (a) with κ= 0.2. The wrapping effect is clearly observable at 0 and 2π; **(c)** Kernel density estimate of data in (a). It shows the sum of the kernel functions after it has been normalized. Notice the peak near 0 is wrapped to 2π. This is a result of the kernels being circular distributions. If a non-circular kernel such as a Gaussian were used, the distribution would be underestimated in this region. The bandwidth of the kernel (in this case the value of kappa) can be adjusted to make the density estimate more sensitive or less sensitive to high-frequency structure.

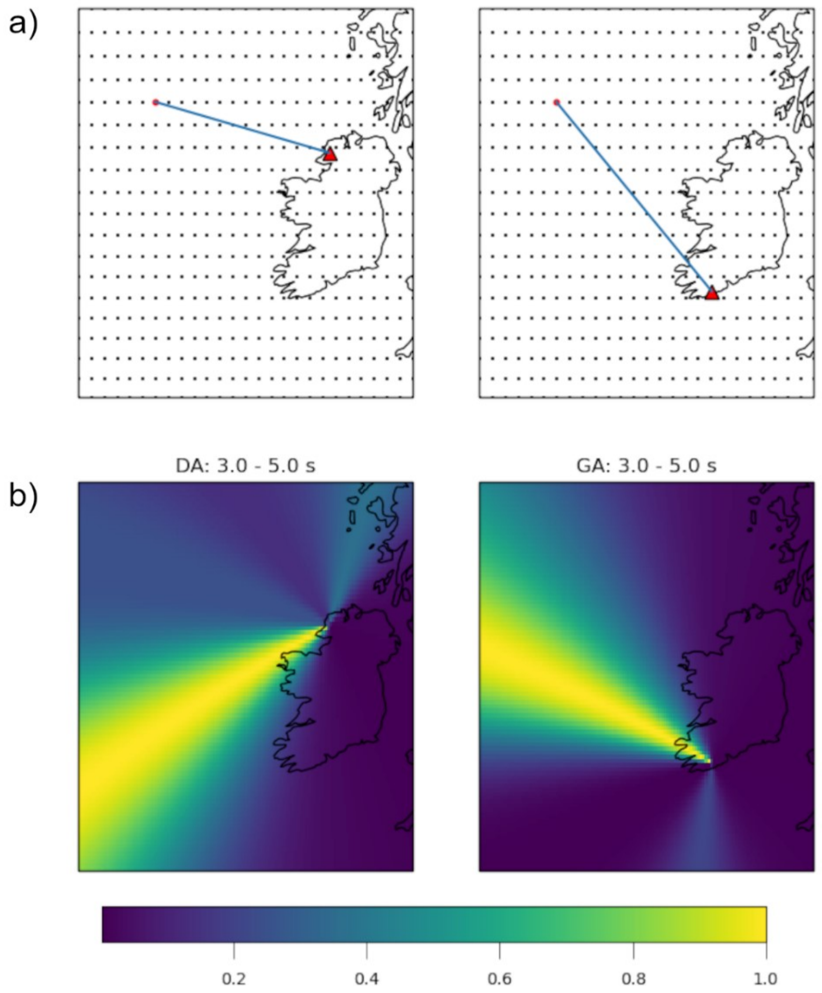

Supplementary Figure 28 – Construction of the source distribution maps. a) Examples of the grid used to visualize the distributions for each array. Only every 5th node is shown for clarity; **b)** Examples of the relative distributions of back azimuth in the period band 3-5s for each array over the analysis period (March 2016-March 2017), projected onto the spatial grid in a).

SUPPLEMENTARY TABLES

	Vp (m/s)	Vs (m/s)	ρ (kg/m^3)	Qp	Qs
**Water**	1500	-	1000	-	-
**Sediments**	2000	610	1910	80	40
	2500	980	2090	140	70
	3000	1410	2220	208	104
	4000	2280	2390	380	190
**Crust**	5000	3010	2540	580	290
	6000	3550	2720	810	405
	6500	3770	2830	920	460
	6900	3950	2940	1020	510
**Mantle**	7800	4460	3220	1350	675

Supplementary Table 1 – Detailed parameters used in the 3D models. The water column is defined with an acoustic wave velocity of 1500 m/s, the sedimentary layers are defined with Vp velocities between 2000 and 4000 m/s, the crustal layers are defined between 5000 and 6900 m/s and the mantle layer is defined with a velocity of 7800 m/s. Shear velocity (Vs), density and anelastic attenuation values are implemented based on Vp values using empirical relations[6,7].

SUPPLEMENTARY REFERENCES

1. Herrmann, R. B. Computer Programs in Seismology: An Evolving Tool for Instruction and Research. *Seismol. Res. Lett.* **84**, 1081–1088 (2013).
2. Konno, K. & Ohmachi, T. Ground-motion characteristics estimated from spectral ratio between horizontal and vertical components of microtremor. *Bull. Seismol. Soc. Am.* **88**, 228–241 (1998).
3. Beyreuther, M. *et al.* ObsPy: A Python Toolbox for Seismology. *Seismol. Res. Lett.* **81**, 530–533 (2010).
4. Rost, S. & Thomas, C. Array seismology: Methods and applications. *Rev. Geophys.* **40**, (2002).
5. Wathelet, M., Jongmans, D., Ohrnberger, M. & Bonnefoy-Claudet, S. Array performances for ambient vibrations on a shallow structure and consequences over V s inversion. *J. Seismol.* **12**, 1–19 (2008).
6. Brocher, T. M. Empirical relations between elastic wavespeeds and density in the Earth's crust. *Bull. Seismol. Soc. Am.* **95**, 2081–2092 (2005).
7. Brocher, T. M. Key elements of regional seismic velocity models for long period ground motion simulations. *J. Seismol.* **12**, 217–221 (2008).
