# Supplementary material for: How deep ocean-land coupling controls the generation of secondary microseism Love waves
Source: Nat Commun. 2021 Apr 20;12:2332. doi: 10.1038/s41467-021-22591-5 (PMC8058104; doi:10.1038/s41467-021-22591-5)
Supplement: Supplementary file 3 — Description of Additional Supplementary Files [file 41467_2021_22591_MOESM3_ESM.pdf]

Description of Additional Supplementary Files

File Name: Supplementary Movie 1

Description: Animation of the vertical wavefield associated with Supplementary Figure 6

File Name: Supplementary Movie 2

Description: Animation of the transverse wavefield associated with Supplementary Figure 7

File Name: Supplementary Movie 3

Description: Animation of the vertical wavefield associated with Supplementary Figure 7

File Name: Supplementary Movie 4

Description: Animation of the transverse wavefield associated with Supplementary Figure 8

File Name: Supplementary Movie 5

Description: Animation of the vertical wavefield associated with Supplementary Figure 8

File Name: Supplementary Movie 6

Description: Animation of the transverse wavefield associated with Supplementary Figure 10

File Name: Supplementary Movie 7

Description: Animation of the vertical wavefield associated with Supplementary Figure 10

File Name: Supplementary Movie 8

Description: Animation of the transverse wavefield associated with Supplementary Figure 11

File Name: Supplementary Movie 9

Description: Animation of the vertical wavefield associated with Supplementary Figure 11
